# Poroid hidradenoma of the eyelid: a case report

**DOI:** 10.3389/fopht.2026.1806872

**Published:** 2026-05-07

**Authors:** Christian Nieves-Rios, José J. López-Fontanet, Juan Serrano-Olmo, Joseph Campbell

**Affiliations:** 1 Department of Ophthalmology, School of Medicine, University of Puerto Rico, San Juan, Puerto Rico; 2 Department of Pathology, San Pablo Pathology Group, Bayamon, Puerto Rico

**Keywords:** case report, eccrine, eyelid, hidradenoma, poroid

## Abstract

**Background:**

Poroid hidradenoma (PH) is a rare, benign tumor arising from eccrine sweat glands. It typically presents as a painless and slow-growing subcutaneous lesion in individuals in their sixth to seventh decades of life. Histologically, PH demonstrates neoplastic poroid cells with solid and cystic structures confined to the dermal layer, distinguishing it from other types of eccrine tumors such as eccrine poroma, hidroacanthoma simplex, and dermal duct tumor. It predominantly involves regions abundant in eccrine glands, yet reports involving the eyelids are scarce.

**Case presentation:**

We report a case of an 88-year-old woman with a 2-year history of a non-painful, enlarging mass on her left upper eyelid, associated with irritation and itchiness. Examination revealed a skin-colored, cystic lesion with associated madarosis and minimal telangiectasia. The patient underwent excisional biopsy of the lesion. Histopathological analysis confirmed the diagnosis of PH, characterized by well-circumscribed solid and cystic components with a pseudocapsule. There was no evidence of recurrence at the 3-month follow-up visit.

**Conclusion:**

This case highlights the rare occurrence of PH in the eyelids, emphasizing the diagnostic challenges associated with adnexal tumors in atypical locations. Surgical excision remains the standard treatment, and awareness of ocular manifestations of PH is essential for accurate diagnosis and management by ophthalmologists.

## Introduction

Poroid hidradenoma (PH) is a rare, benign adnexal tumor originating from eccrine sweat glands (1). It mainly affects individuals in their sixth to seventh decades of life; however, it has been reported across a diverse range of age groups and with no sex or ethnic predilection (1, 2). Generally, PH presents as a painless, slow-growing, well-defined subcutaneous lesion, predominantly in regions abundant in eccrine glands, such as the head, neck, and limbs (1, 2), but it is rarely reported in the eyelids. Histologically, PH demonstrates neoplastic poroid cells with solid and cystic structures confined to the dermal layer, distinguishing it from other types of eccrine tumors such as eccrine poroma, hidroacanthoma simplex, and dermal duct tumor (1, 3).

Clinical differentiation between the variants of poroid neoplasms is challenging. Therefore, the diagnosis of PH is primarily histological, and the treatment involves surgical excision (1). Although benign, excising these tumors is advised to prevent any possible malignant transformation. We present an occurrence of PH on the eyelid. Despite its benign nature, the rarity and unusual location of PH contribute to the challenges of its diagnosis and management.

## Case presentation

An 88-year-old woman presented with a 2-year history of an enlarging, non-painful mass on her left upper eyelid. The patient reported associated irritation and itchiness but denied any purulent discharge, ulceration, or bleeding. Her medical history included a previous excision of a lesion on the same eyelid 10 years prior at another facility. However, no pathology report was available.

**Figure 1:**
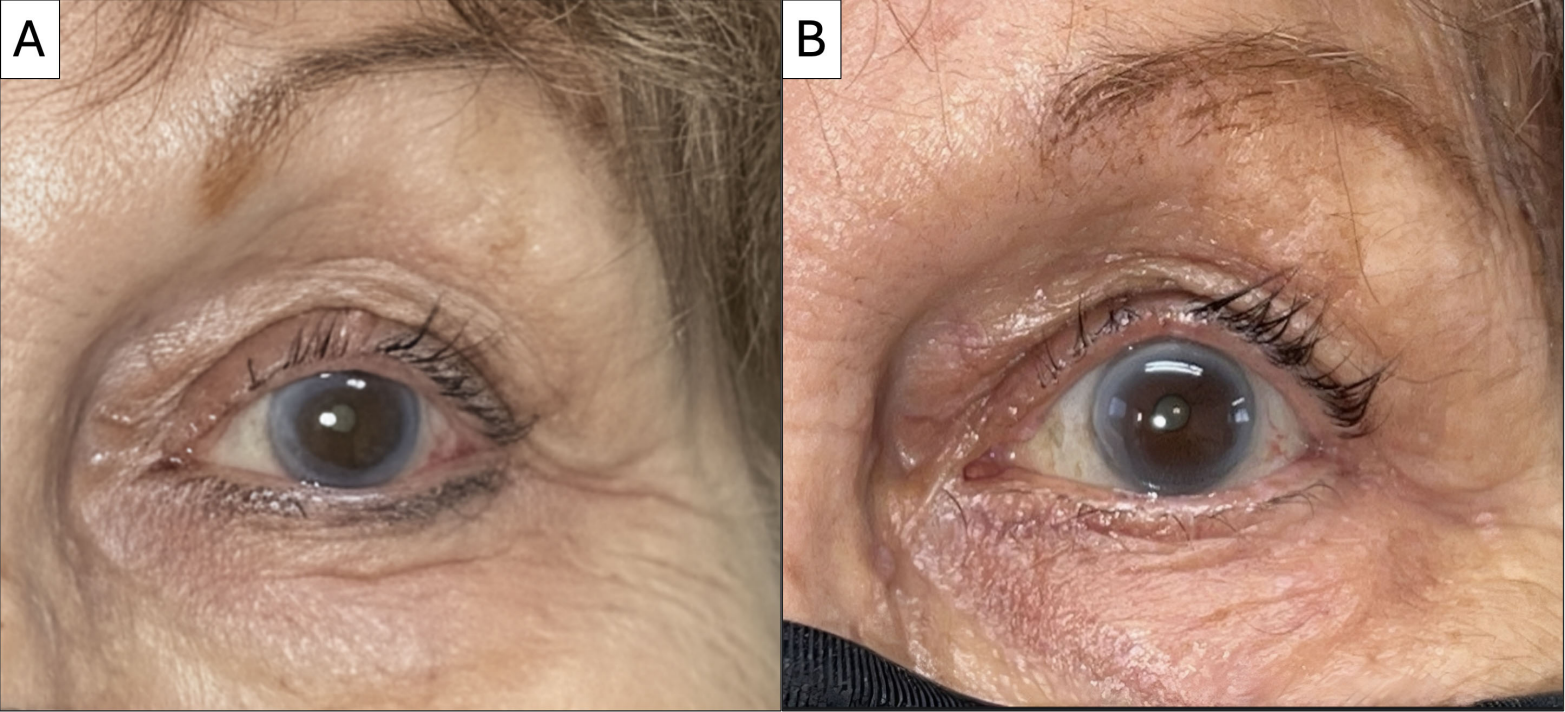
External photograph at presentation and after surgical excision. **(A)** Image shows skin-colored cystic lesion on the central aspect of the left upper eyelid. **(B)** Post-operative image demonstrates no recurrence of the lesion at the 3-month follow-up visit.

On examination, her visual acuity was 20/25 in the right eye and 20/40 in the left eye. Intraocular pressures were normal, and she demonstrated complete extraocular movements bilaterally. The external examination revealed a skin-colored cystic lesion on the central aspect of the left upper eyelid, accompanied by madarosis and minimal telangiectasia, as shown in **Figure 1A**. There was no evidence of proptosis or ptosis, and the anterior segment examination was within normal limits.

**Figure 2:**
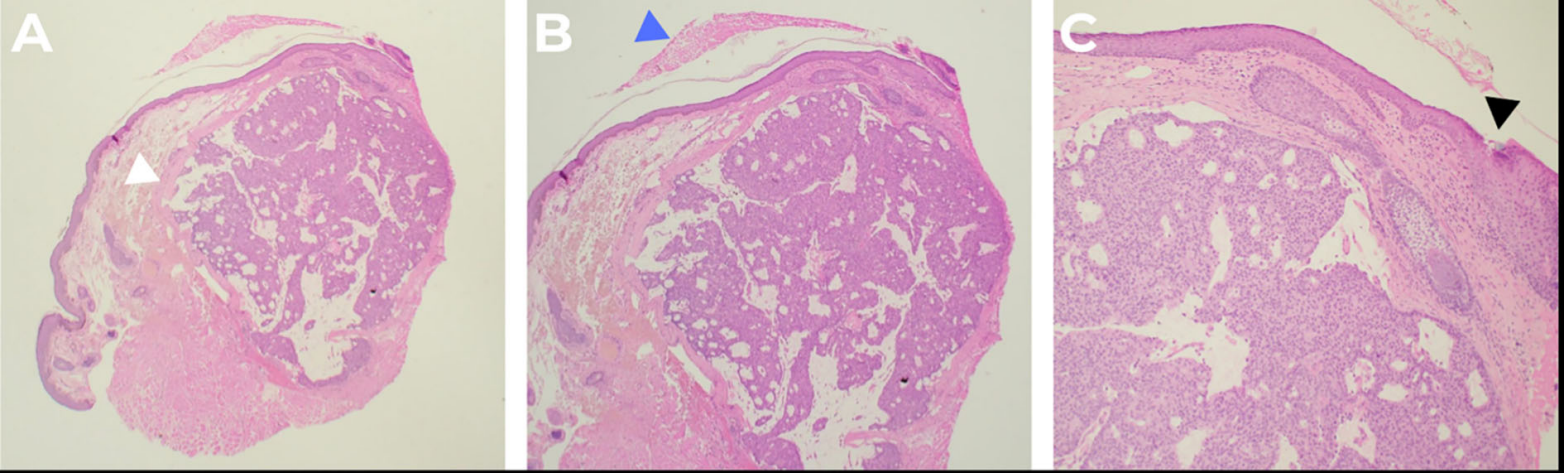
Low-power microscopic findings. **(A)** At 2× magnification, a well-circumscribed tumor is observed, composed of both solid and cystic components, exhibiting a pseudocapsule growing toward the dermis (indicated by a white arrow). The tumor shows cribriform formation with multiple lumens and solid areas. **(B)** At 4× magnification, the scale crust over the lesion is visible (indicated by a blue arrow). The image highlights the external surface characteristics of the tumor. **(C)** At 10× magnification, a hair follicle can be seen (indicated by a black arrow). This higher magnification image allows for the detailed observation of the tumor’s interaction with adjacent structures.

**Figure 3:**
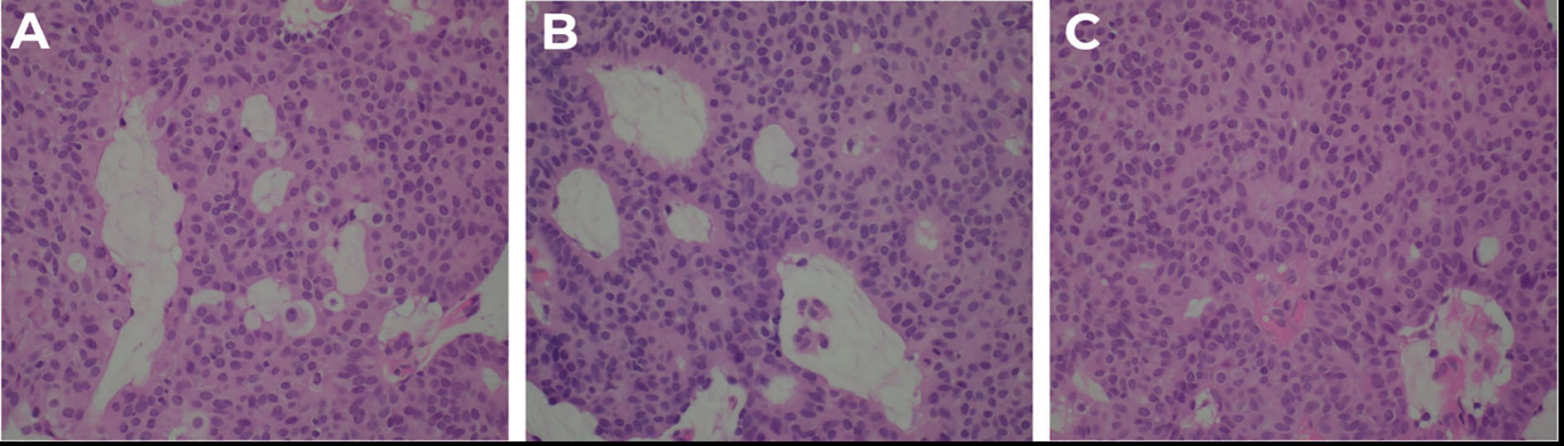
High-power microscopic findings. **(A–C)**. At 40× magnification, smaller polygonal cells with pale eosinophilic cytoplasm and vesicular nuclei containing eosinophilic nucleoli and nuclear grooves are observed. The images show solid areas with evident mitotic activity. Central secretion and glandular formation are visible, with basal nuclei distribution.

An excisional biopsy of the left upper eyelid lesion was subsequently performed without complications. The lesion measured 5.0 mm × 4.0 mm × 3.0 mm at the time of excision. Histopathological examination revealed a well-circumscribed tumor with both solid and cystic components, exhibiting a pseudocapsule that extended toward the dermis. Cribriform formation with multiple lumens and solid areas is shown in **Figures 2A–C**. High-power microscopic views revealed small polygonal cells with pale eosinophilic cytoplasm, vesicular nuclei containing eosinophilic nucleoli, and nuclear grooves. Areas of mitotic activity, central secretion, and glandular formation with basal nuclei distribution were also observed, as shown in **Figures 3A–C**. The morphological features were consistent with PH of the eyelid, with clear margins and no characteristics suggestive of malignancy. At the 3-month follow-up visit, no evidence of recurrence was observed, as shown in **Figure 1B**.

## Discussion

Poromas are cutaneous adnexal tumors originating from sweat glands, commonly found on the palms and soles, or on the head and neck (1). The former are mostly of eccrine origin, while the latter are typically apocrine (4–6). Clinically, poromas exhibit diverse features and can be challenging to distinguish from other adnexal tumors, such as melanocytic nevus, hemangioma, pyogenic granuloma, wart, and cyst (4).

PH has been described as the rarest of the four variants of poroid neoplasms (1). These lesions are more commonly seen in elderly patients, yet reports in younger patients have also been described (1). PH is characterized by a single or multilobulated nodule located within the dermis without connection to the epidermis (1). Signs of PH include a slow-growing, painless subcutaneous mass with overlying normal pink skin or a dark and/or bluish hue (1). Immunohistochemical analysis may be useful for equivocal cases, and diagnosis may be supported by positive staining for markers such as epithelial membrane antigen, carcinoembryonic antigen, and low-molecular-weight cytokeratins (1). Patients may be asymptomatic or may complain of itching, pain, paresthesia, bleeding, or ulceration (4).

Malignant transformation of PH is rare, occurring in less than 1% of cases (7). Nonetheless, surgical excision with clear margins (2–3 mm) remains the standard treatment, and recurrence is uncommon (1). Initial close follow-up may be suitable for recurrent or atypically located lesions, with the possibility of extending to biannual or annual evaluations. In our case, the patient demonstrated resolution of the lesion with no recurrence. However, follow-up of our patient was limited, and long-term prognosis may be uncertain. Moreover, considering the history of a preceding lesion in the same eyelid, the possibility of recurrence due to an incomplete excision may be considered. However, confirmation was not possible due to the absence of the previous biopsy result.

Reports of poromas on the eyelids are limited, with the majority being described as eccrine poromas (8–12). To our knowledge, poroid hidradenoma of the eyelid has not been previously reported in the English literature, underscoring its rarity in the ocular adnexa compared with more common head, neck, and limb sites. Compared to reported eyelid eccrine poromas, which are often surface-connected and present at variable ages, our case was fully dermal, with both cystic and solid components. It also presented as a slow-growing lesion in an elderly patient, similar to general poroid hidradenoma presentations but unique in location. Therefore, increasing awareness of the ocular manifestation of this histological subtype of poromas is essential for ophthalmologists to ensure accurate diagnosis and management.

## Conclusions

This case highlights the occurrence of PH in the eyelid, emphasizing the diagnostic challenges associated with adnexal tumors in atypical locations. Surgical excision remains the standard treatment, yielding excellent clinical results and a low risk of recurrence. Awareness of ocular manifestations of PH is essential for accurate diagnosis and management by ophthalmologists.

## Data Availability

The original contributions presented in the study are included in the article/supplementary material. Further inquiries can be directed to the corresponding author.
